# Electromyography and kinematics data of the hand in activities of daily living with special interest for ergonomics

**DOI:** 10.1038/s41597-023-02723-w

**Published:** 2023-11-20

**Authors:** Alba Roda-Sales, Néstor J. Jarque-Bou, Vicent Bayarri-Porcar, Verónica Gracia-Ibáñez, Joaquín L. Sancho-Bru, Margarita Vergara

**Affiliations:** Department of Mechanical Engineering and Construction, Universitat Jaume I, 12071 Castelló de la Plana, Spain

**Keywords:** Muscle, Skeleton, Databases

## Abstract

This work presents a dataset of human hand kinematics and forearm muscle activation collected during the performance of a wide variety of activities of daily living (ADLs), with tagged characteristics of products and tasks. A total of 26 participants performed 161 ADLs selected to be representative of common elementary tasks, grasp types, product orientations and performance heights. 105 products were used, being varied regarding shape, dimensions, weight and type (common products and assistive devices). The data were recorded using CyberGlove instrumented gloves on both hands measuring 18 degrees of freedom on each and seven surface EMG sensors per arm recording muscle activity. Data of more than 4100 ADLs is presented in this dataset as MATLAB structures with full continuous recordings, which may be used in applications such as machine learning or to characterize healthy human hand behaviour. The dataset is accompanied with a custom data visualization application (ERGOMOVMUS) as a tool for ergonomics applications, allowing visualization and calculation of aggregated data from specific task, product and/or participants’ characteristics.

## Background & Summary

The complexity of the human hand, with 25 main degrees of freedom (DoF) controlled by more than 30 muscles, provides the required ability to perform activities of daily living (ADLs). Understanding the relationship between hand movements and forearm muscular activation during ADLs is challenging, and may be useful for several applications such as improving control of prosthetic hands^1^, developing more realistic hand models^2^, or improving hand rehabilitation methods^3^. Nevertheless, for these purposes a large amount of kinematic and muscular activation data is needed. In this sense, several researchers^4^ have pointed out the importance of high-quality open-access datasets of grasping data, while also highlighting the need to compile, classify and standardize these data.

Product designers and ergonomists can also benefit from these databases. Product ergonomics assessment is usually limited to studying qualitative kinematic parameters such as grasp type used or hand contacting areas, and grip strength^5^. Analysing kinematic and muscular activation parameters such as median or extreme postures used by participants with varied characteristics when performing different tasks with products with different characteristics would provide important information regarding the effect of product design characteristics, contributing to the design of more inclusive products.

Although some datasets of hand kinematics during task performance are available in the literature^6–10^, as well as forearm EMG datasets, very few datasets exist with simultaneously recorded kinematics and EMG^11–15^, and they present several weaknesses. The main weakness is that the tasks recorded are not representative of ADLs^11,12,14,15^, being mainly grasping movements or static hand postures. Moreover, the kinematic data are sometimes presented as raw data instead of anatomical angles^11^, or have been collected with methods that are not the most reliable^12^.

To tailor a dataset representative of human hand behaviour (therefore allowing to characterise human hand function from a clinical point of view, to apply artificial intelligence techniques or to provide data to product designers, among others), datasets must contemplate a wide range of tasks representative of ADLs, elementary actions (holding, twisting, pouring, etc.) and grasp types, as several studies evidence that the task performed affects hand posture^16^. Another key aspect is considering products with different design characteristics, as aspects such as handle diameters have been observed to affect functional range of motion^17^, and aspects such as additional handles, thickened handles or bent handles have been found to produce specific effects in extreme postures and ranges of motion^18^. Similarly, handle diameter, weight and object position have been observed to affect forearm muscle activity^19–21^. Furthermore, several studies evidence an effect on grasp type selected when using products with different design characteristics^22–24^. Apart from these effects of the product itself, a representative sample of participants has to be recruited, considering participants’ gender, age and hand length. Finally, some technical aspects have to be considered, such as continuous recording of data (especially in ADLs requiring product manipulation, where the complexity of the tasks makes hand posture more variable), using the most appropriate units of measure depending on the purpose of the data collected, or providing data for both the dominant and non-dominant hands.

In this paper we present the MOVMUS-UJI Dataset, which contains a total of 4186 recordings with hand anatomical angles and forearm muscular activation while performing activities of daily living, along with tagged characteristics of products used, tasks performed and participants. Data are presented as MATLAB structures, accompanied with a custom data visualization application, ERGOMOVMUS, to ease the use of the data for ergonomists. The main contribution of this dataset compared to others is its synchronization of hand kinematics and forearm muscle activation with tagged information regarding the subject’s and task’s characteristics, and product design features, allowing more specific data analyses focusing on certain task/product characteristics. One of the strengths of the dataset is the variety of products used (105 products) and the variety of tasks performed requiring different elementary actions (161 different tasks, divided into 614 elementary tasks), and different grasp types, performance heights and product orientations. It is also worth mentioning that the sample of participants was selected so as to be representative of the healthy adult population (with a controlled proportion of ages and genders). Another important strength is the presentation of data, both available as a MATLAB/GNU Octave data structure (*.mat)* containing the full continuous recordings, and also a statistical summary of the recordings (5^th^, 50^th^ and 95^th^ percentiles) in a spreadsheet file (in.xlsx or.ods) accompanied with a custom data visualization application. The data structure may be useful for users experienced in coding, having a lot of potential for applications such as machine learning, as it contains complete continuous recordings. The statistical summary of these recordings, along with the data visualization application, provides a more intuitive way to access statistical data. This can be particularly valuable for specialists such as clinicians or product designers when characterizing healthy human hand behaviour or optimizing product ergonomics, among other applications. This dataset is accompanied with a usage guide, which contains detailed information regarding the environment, tasks, objects, data acquisition system, visualization application and file structure details. The data presented herein follow standard rules: kinematic data are provided as anatomical angles following the ISB sign criteria^25^, and muscular activity is presented as a value between 0–1 proportional to participants’ maximum voluntary contraction (MVC).

## Methods

### Study participants

The study consisted of three phases (A, B and C), with 26 right-handed participants (13 males, 13 females) participating in each phase. Only 22 participants took part in all the phases of the experiment, so that the total amount of participants recruited was 30. The mean age was 30.81 ± 11.17 years in phase A and 31.19 ± 11.18 years in phases B and C. Inclusion criteria were gender parity in overall data, right-handedness, age between 20 and 55 years and no reported upper limb pathologies. Recruitment was performed through print advertisements and social media. All participants gave written informed consent before the experiments, which were performed in accordance with the principles of the Declaration of Helsinki. Approval was granted by the Research Ethics Committee with Human Beings (formerly Deontology Committee) of Universitat Jaume I (Spain), reference number CD/31/2019.

### Acquisition setup

#### Motion capture equipment

**Fig. 1:**
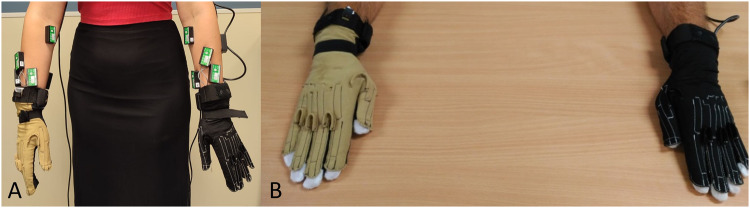
(**A**) Participant equipped with Biometrics sEMG electrodes (LE230) and CyberGlove data gloves. (**B**) Hand reference posture for kinematics recording.

Kinematic data were acquired using two instrumented gloves (CyberGlove Systems LLC): a CyberGlove II on the right hand and a CyberGlove III on the left hand (Fig. 1a). Each of these gloves has 18 strain gauges, allowing to determine the anatomical angles of the underlying joints. The angle rotated by each joint with respect the reference posture (hands resting flat on a table, with the fingers and thumb together, and the middle fingers aligned with the forearms, see Fig. 1b) is then obtained from these signals, according to a previously validated calibration protocol^26^.

#### sEMG equipment

**Fig. 2:**
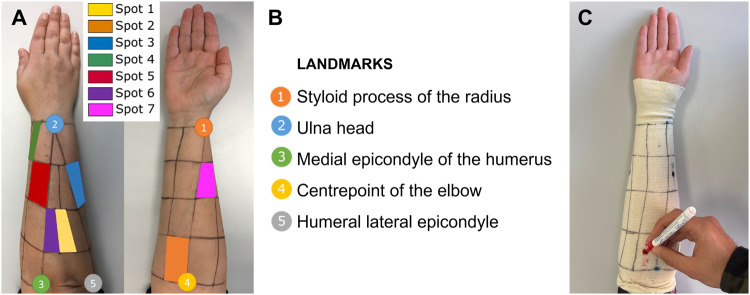
(**A**) Grid and spot areas selected for the sEMG recordings. (**B**) Five anatomical landmarks used to draw the grid. (**C**) Tubigrip with the grid defining the spots location. The signals from these seven spots are related to seven different movements according to previous work^27^. Spot 1: wrist flexion and ulnar deviation (WF_UD); spot 2: wrist flexion and radial deviation (WF_RD); spot 3: digit flexion (DF); spot 4: thumb extension and abduction/adduction (TM); spot 5: finger extension (FE); spot 6: wrist extension and ulnar deviation (WE_UD); spot 7: wrist extension and radial deviation (WE_RD).

Muscle activity was recorded using a Biometrics DataLITE wireless recording unit (Biometrics Ltd.) at a sampling frequency of 1000 Hz. Seven integral dry reusable wireless sEMG electrodes (LE230) were used for each forearm. Electrodes were placed in the centre of seven representative spot areas of the right forearm, according to previous work^27^, and were set out in longitudinal direction (Figs. 1, 2), following the SENIAM recommendations^28^. To locate these seven spot areas, a Tubigrip with a grid defining the spots location was put on the subject’s forearm, placing it using five easily identifiable anatomical landmarks^27^ (Fig. 2). These seven spots were chosen to be representative of all available muscle activity of the whole forearm, according to previous works^27^.

#### Environment

**Fig. 3:**
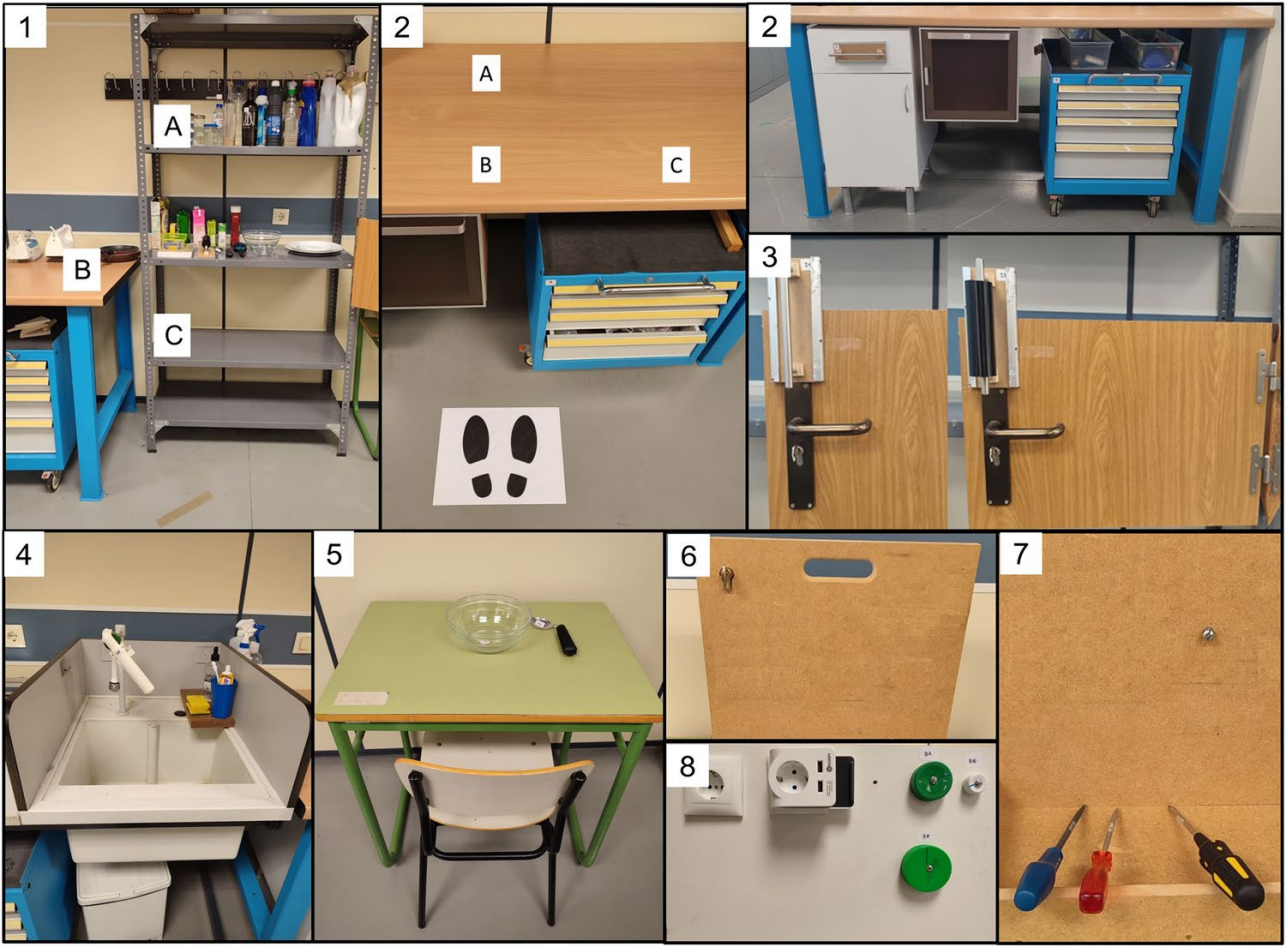
Different scenarios of the experiment. Scenarios: a table and shelves at different heights (1), a table (2), a door with exchangeable handles (3), a sink (4), a desk (5), a lock on a panel (6), a screw in a wooden wall (7) and electrical sockets and turning buttons on a panel (8).

The tasks were performed in a laboratory environment that simulated home areas. The scenarios (Fig. 3) consisted of: a table and shelves at different heights (Scenario 1), a table (Scenario 2), a door with exchangeable handles (Scenario 3), a sink (Scenario 4), a desk (Scenario 5), a lock on a panel (Scenario 6), a screw in a wooden wall (Scenario 7) and electrical sockets and turning buttons on a panel (Scenario 8).

#### Products

**Fig. 4:**
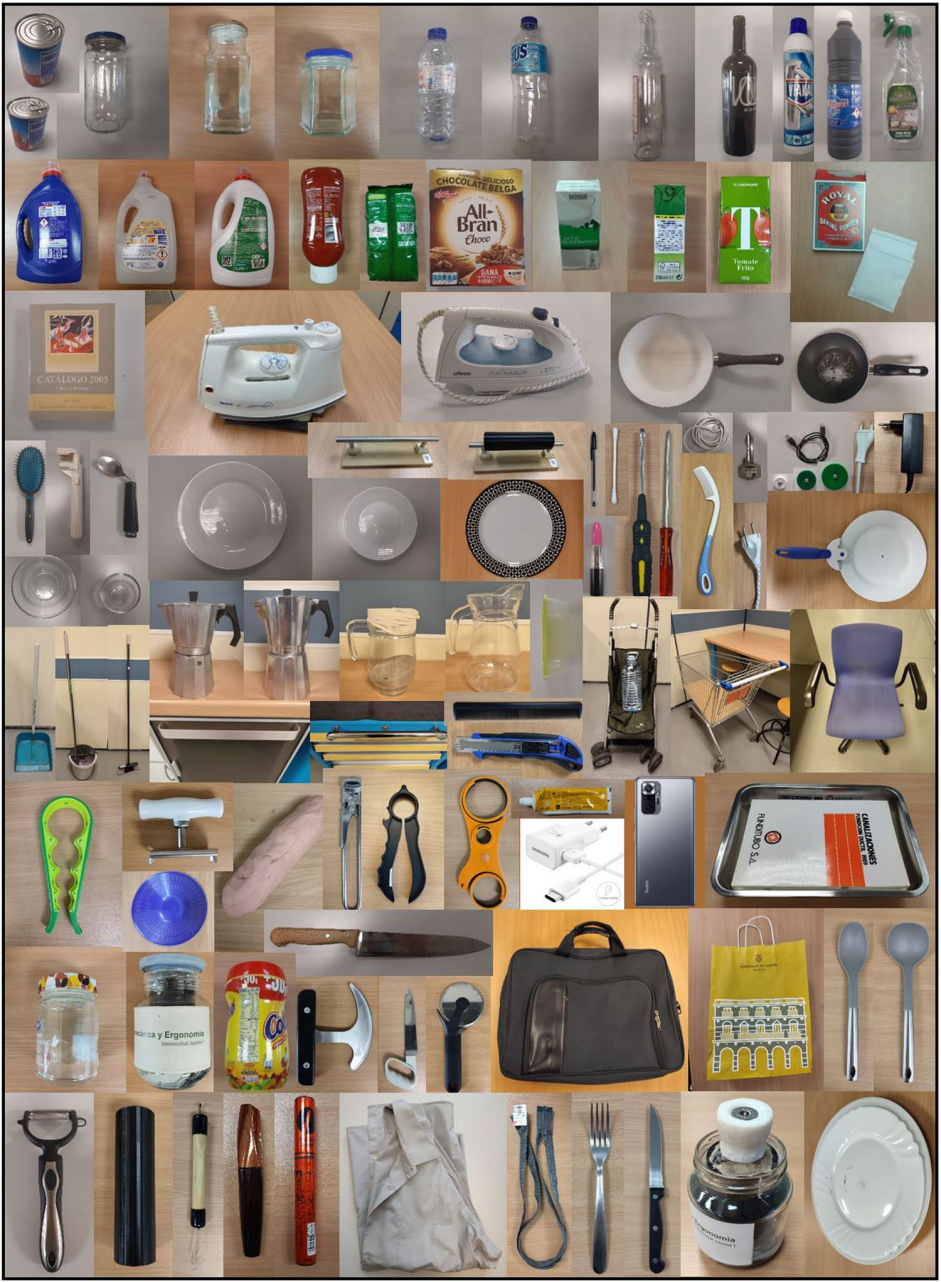
Overview of the products used to perform the tasks.

The 105 products used in the recorded tasks were selected to be representative of the wide variety of products commonly used to perform ADLs. Thus, different product types were selected: bottles, cans, jars, houseware, food packaging, cutlery, assistive devices, self-care and cleaning products, among others. Furthermore, they were selected to cover different weights, dimensions and section shapes (further information regarding their characteristics can be found in the guide attached to the dataset). All the products used were real, except in the task of peeling a cucumber (the cucumber was replaced with a 3D printed cucumber) and in tasks requiring cutting food (play dough was used instead of food). The location of the products in each scenario is detailed in the guide attached to the dataset. Figure 4 shows an overview of the products used.

### Acquisition protocol

The main dimensions of participants’ hands were measured before wearing the instrumented gloves. Furthermore, participants’ forearm hair was removed and skin cleaned with alcohol before placing the sEMG sensors in the forearm. Then, a kinematic reference posture (hands lying flat on a table with fingers and thumbs close together, with middle fingers aligned with forearms) was recorded before performing all the experiment tasks, and was considered zero for all the anatomical angles.

**Fig. 5:**
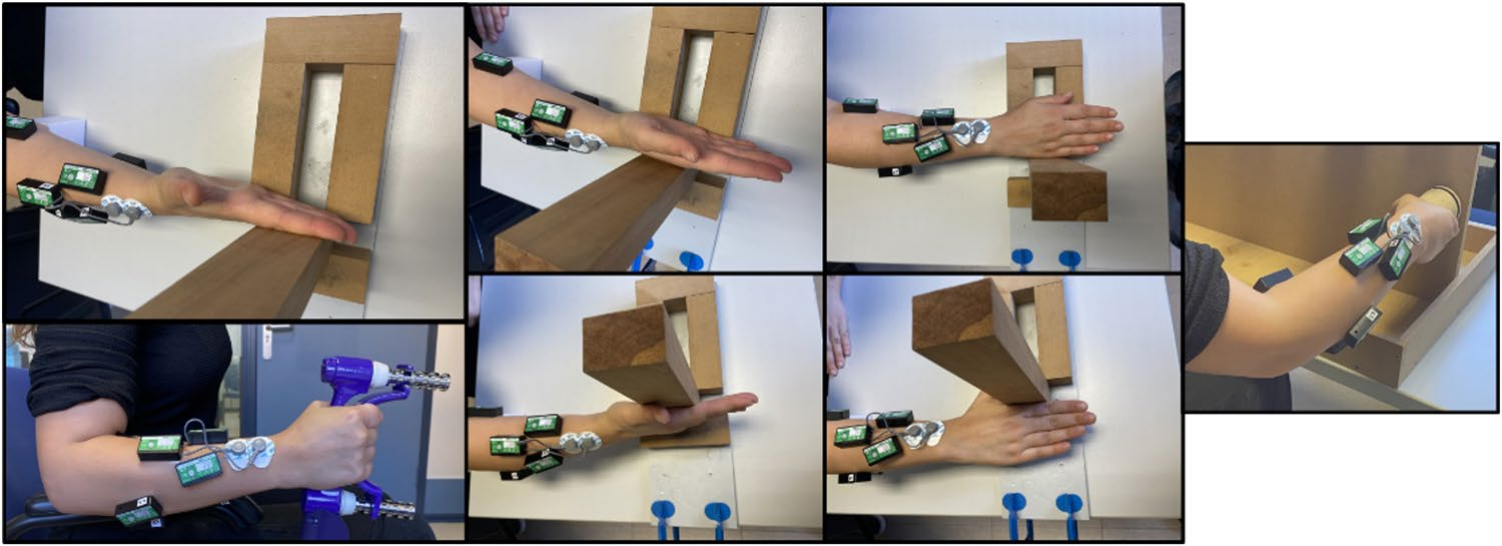
Seven MVC records for the normalization of the muscle activity signal. From left to right: flexion and extension of the fingers, flexion and extension of the wrist, ulnar and radial deviation of the wrist, and pronation of the forearm.

To normalize muscular activation signal, seven records of maximum voluntary contraction (MVC) were performed: flexion and extension of the fingers, flexion and extension of the wrist, ulnar and radial deviation of the wrist, and pronation of the forearm (Fig. 5). Participants were asked to take a comfortable posture and exert maximum effort without the help of muscles other than those of the forearm and hand.

#### Signal synchronisation

The CyberGlove kinematic data and sEMG records were synchronised using custom data acquisition software especially designed for this purpose, matching the initial and final instants of each record. This acquisition software was developed in C++, synchronising glove and sEMG by using the SDK libraries of the CyberGlove and Biometrics devices.

#### Recorded tasks

The experiment was performed in three different phases: A, B and C. In phase A, the tasks performed only required using the right hand with cylindrical, lateral pinch and lumbrical grasp types. In phase B, the tasks only required using the right hand with oblique, special pinch, hook, intermediate and five-finger pinch grasp types. Tasks in phase C required using both hands (bimanual tasks) with all the previously mentioned grasp types. Order of tasks was randomized for each participant. One complete task was performed during each continuous run of data recording, during which the operator marked specific events that were later used to separate the task’s component elementary tasks, e.g. holding, pouring, twisting, etc. Therefore, phase A was composed of 79 tasks (342 elementary tasks), phase B consisted of 33 tasks (128 elementary tasks), and phase C of 49 tasks (144 elementary tasks). Further information regarding the elementary tasks considered in each recording can be found in the guide attached to the dataset. An example for one task can be seen in Table 1.

**Table 1:** Elementary tasks into which task T = 111 is divided.

T	ET	PR	SC	ELEMENTARY TASK
111	55	9	1	Move closer to take the object from the top shelf (reaching phase)
	56			Take the object from the top shelf. Leave it on the kitchen top
	57			Release the object and return to P1 (release phase)
	58			Move closer to take the object from the kitchen top (reaching phase)
	59			Take the object from the kitchen top. Leave it on the bottom shelf
	60			Release the object and return to P1 (release phase)

Columns containing T (ID of the task), ET (ID of the elementary task), PR (ID of the products used during the task), SC (ID of the scenario where the task is performed).

The participants were given clear instructions about how to perform each task, and they were told to start and end the task in the same posture: hands lying relaxed at both sides of the body for tasks performed in a standing posture, and hands lying relaxed on the table when sitting (initial posture for each task is specified in the guide attached to the dataset). While carrying out each task, the operator marked specific events that were later used to separate the different elementary tasks.

For each task, participants were given clear instructions on the elementary tasks to be performed and on the grasp types to be used by each hand. Table 1 illustrates the instructions given to participants as well as labelling of tasks and elementary tasks in the dataset.

### Signal processing

#### Angles calculation

**Fig. 6:**
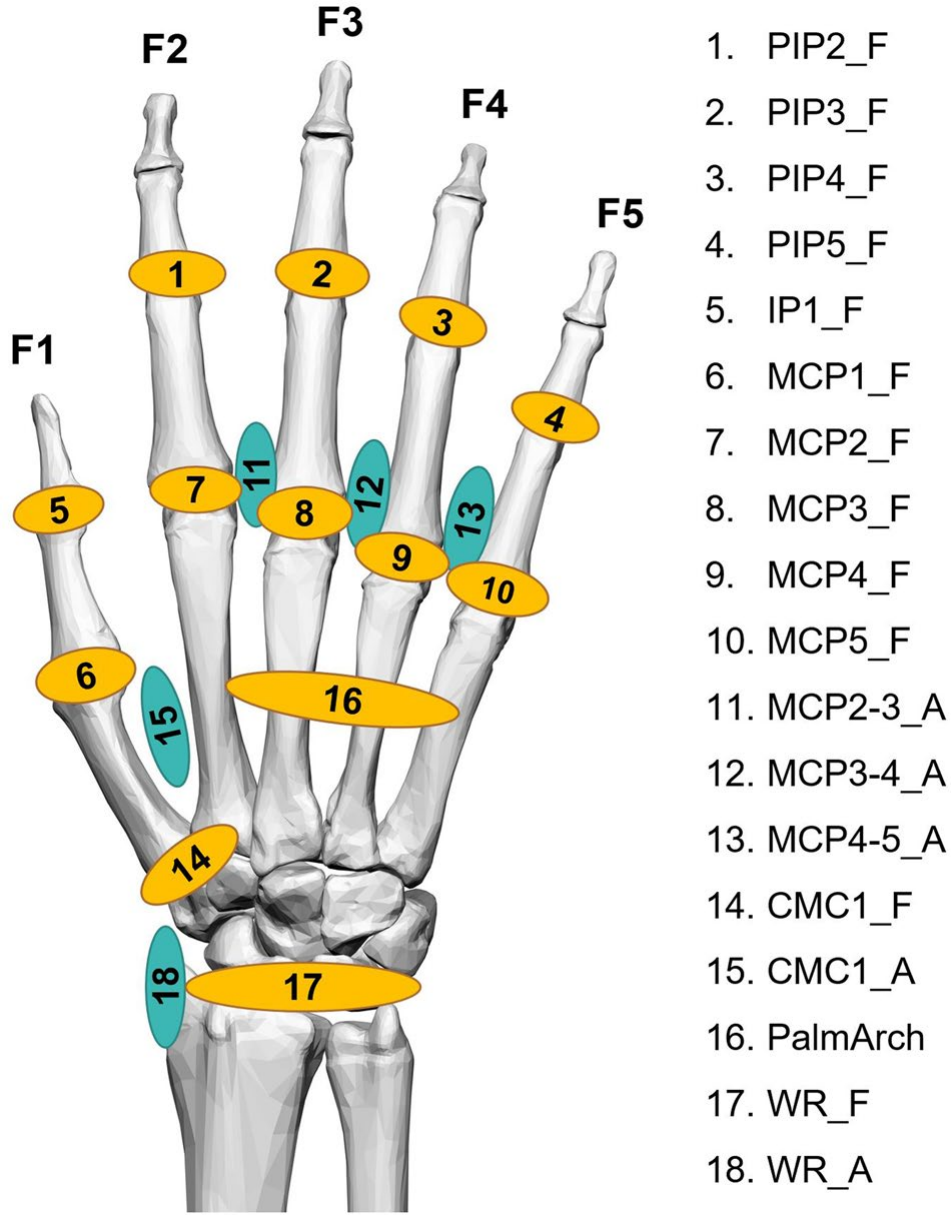
Recorded anatomical angles. Nomenclature: _F for flexion (in yellow), _A for abduction (in turquoise); digits 1 to 5. Joints: IP (interphalangeal joint), PIP (proximal interphalangeal joints), MCP (metacarpophalangeal joints), CMC (carpometacarpal joints), PalmArch (palmar arch resulting from flexion/extension of carpometacarpal joints of ring and little fingers), WR (wrist).

Joint angles were computed from raw glove data following the calibration protocol proposed in previous works^26^. This protocol includes the determination of gains and also some corrections to avoid cross-coupling effects for specific anatomical angles. The joint angles obtained according to this protocol are shown in Fig. 6:

#### sEMG signal processing

To compute muscle activity, sEMG records were first normalised by dividing them by the maximal values from any record (7 MVCs and all tasks) measured for each subject. In this way, sEMG activity from the same spot of different individuals or sEMG activity between different spots can be compared. Afterwards, the normalised recordings were resampled to 100 Hz to present synchronized data with angles.

#### Data splitting

Each task recorded was separated into different elementary tasks as detailed in the dataset guide by using the labelling performed by the operator while recording the data.

#### Filtering

Kinematic data were filtered with a 2^nd^-order two-way low pass Butterworth filter with a cut-off frequency of 5 Hz. The sEMG data were filtered with a fourth-order bandpass filter between 25–500 Hz, rectified, filtered by a fourth-order low-pass filter at 8 Hz, and smoothed by Gaussian smoothing^29^.

#### Outliers and missing signal during recordings

A total of 4186 tasks (15964 elementary tasks) were recorded across all the participants and experiments. Nevertheless, signal was missing during some recordings. Tasks with missing signal were removed, leaving 15823 elementary tasks in the final dataset. Outliers were erased considering the active and functional range of motion of the joints measured in a previous study^30^. Specifically, the 50th percentile of each joint for each elementary task was calculated and those values higher or lower than the mean range of motion values of each joint plus three times its standard deviation were eliminated. During this analysis, a malfunction of the glove gauge recording right palmar arch flexion was detected after performing the experiments. Therefore, to ensure that all data are correct, data of this DoF are not reported in the dataset. In the end, 55 outliers were deleted: 5 cases in CMC1_F, 30 cases in IP1_F, 11 cases in PIP2_F, 7 cases in PIP3_F, 1 case in PIP4_F and 1 case in PIP5_F. Outliers in the thumb joint may be due to a poor fit of the gauge in that finger. Outliers in PIP joints are negative values (around −20°) and may be due also to a poor fit of these gauges for a specific subject, since most cases were found in the same subject, who had a small hand length.

## Data Records

### Data files

Data are presented as several MATLAB structures stored in.mat files: KIN_EMG_DATA.mat, PARTICIPANT_DATA.mat, TASK_DATA.mat and PRODUCT_DATA.mat. The data are accompanied by a guide (.pdf), which provides more detailed information regarding the data series as well as the environment, tasks, products and data acquisition system. The dataset is publicly available to all research community at the Zenodo open repository^31^.

#### KIN_EMG_DATA

Substructure containing all kinematic and sEMG data recorded, classified by task, elementary task and participant. For the kinematic data, the sign criteria for each joint motion were defined as follows:

**PIP(2-5)_F, IP1_F, MCP(1-5)_F:** Flexion + / Extension -

**MCP(2-3, 3-4, 4-5)_A:** Fingers separated + / Fingers together -

**PalmArch:** Flexion + /Extension –

**Fig. 7:**
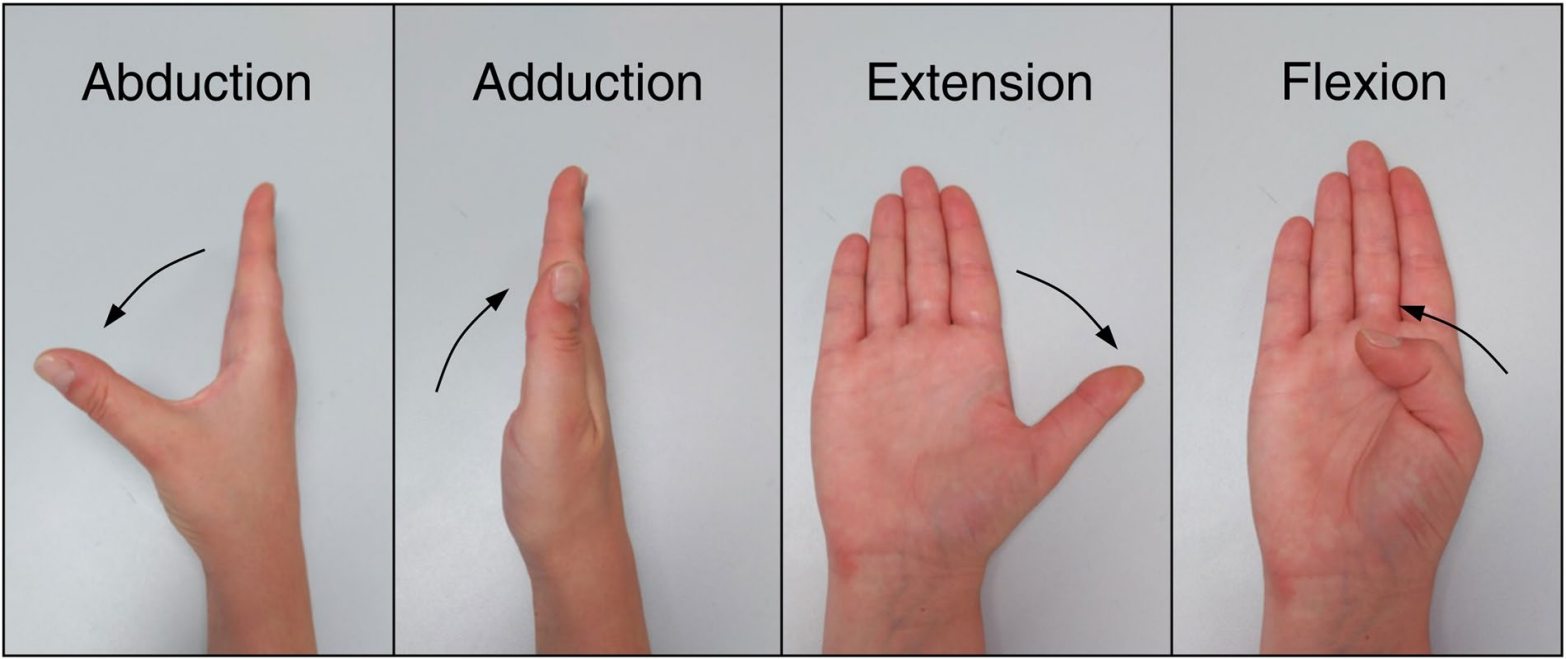
Carpometacarpal joint motion.

**CMC1_F:** Flexion + /Extension – (See Fig. 7)

**CMC1_A:** Abduction + /Adduction - (See Fig. 7)

**WR_F:** Flexion + / Extension -

**WR_A:** Ulnar deviation + /Radial deviation –

Note that movement of thumb CMC joint is complex, and nomenclature used in literature to define these movements is varied^32,33^. We adopted the one used by Brand and Hollister^33^.

For the muscle activity, normalised signal for the seven representative spot areas (Fig. 2) were presented ordered from Spot 1 to 7 (columns 1 to 7 in case of right hand, columns 8 to 14 in case of left hand).

#### PARTICIPANT_DATA

Substructure containing information of the participants recruited (age, gender, hand and forearm length, height, and phases in which the participant was recruited).

#### TASK_DATA

Substructure containing information regarding task characteristics (product grasped/manipulated with each hand, grasp span, use of both hands, product orientation, action performed and height). Table 2 details the variables, their meanings and codification.

**Table 2:** Fields in TASK_DATA substructure.

VARIABLE	VALUE/LABELLING
*T*	ID of the task
*ET*	ID of the elementary task
*PRODUCT_DH*	ID of the product grasped with dominant hand.
*PRODUCT_NDH*	ID of the product grasped with non-dominant hand.
*SPAN_DH*	Span of grasp performed with dominant hand. 0 = None (no product grasped) 1 = Main product span (specified in PRODUCT_DATA) 2 = Secondary product span (specified in PRODUCT_DATA)
*SPAN_NDH*	Span of grasp performed with non-dominant hand. Codified as SPAN DH.
*BIMANUAL*	Hands involved in task: 0 = Non-bimanual (only dominant hand involved) 1 = Bimanual (both hands involved)
*GRASP_DH*	Grasp type performed with dominant hand: 1 = Cylindrical 2 = Lateral pinch 3 = Lumbrical 4 = Oblique 5 = Special Pinch 6 = Hook 7 = Intermediate 8 = Five finger pinch 9 = Free
*GRASP_NDH*	Grasp type performed with non-dominant hand. Codified as GRASP DH.
*ORI_PRODUCT_DH*	Orientation of product grasped with dominant hand: (see Fig. 8) 1 = Vertical 2 = Horizontal (transverse) 3 = Horizontal (longitudinal) 4 = Other (none of the previously mentioned or in motion)
*ORI_PRODUCT_NDH*	Orientation of product grasped with non-dominant hand. Codified as ORI_PRODCUT_DH.
*ACTION_DH*	Action performed with dominant hand: 1 = Reaching 2 = Releasing 3 = Transporting 4 = Holding 5 = Pouring 6 = Pulling 7 = Pushing 8 = Twisting (clockwise) 9 = Twisting (anticlockwise) 10 = Other
*ACTION_NDH*	Action performed with non-dominant hand. Codified as ACTION_DH.
*TASK_HEIGHT*	Height of performance of the task: 1 = High-Median (above shoulder) 2 = Median (between shoulder and hips) 3 = Median-Low (below hips)

Abbreviations: Dominant hand as DH, non-dominant hand as NDH.

**Fig. 8:**
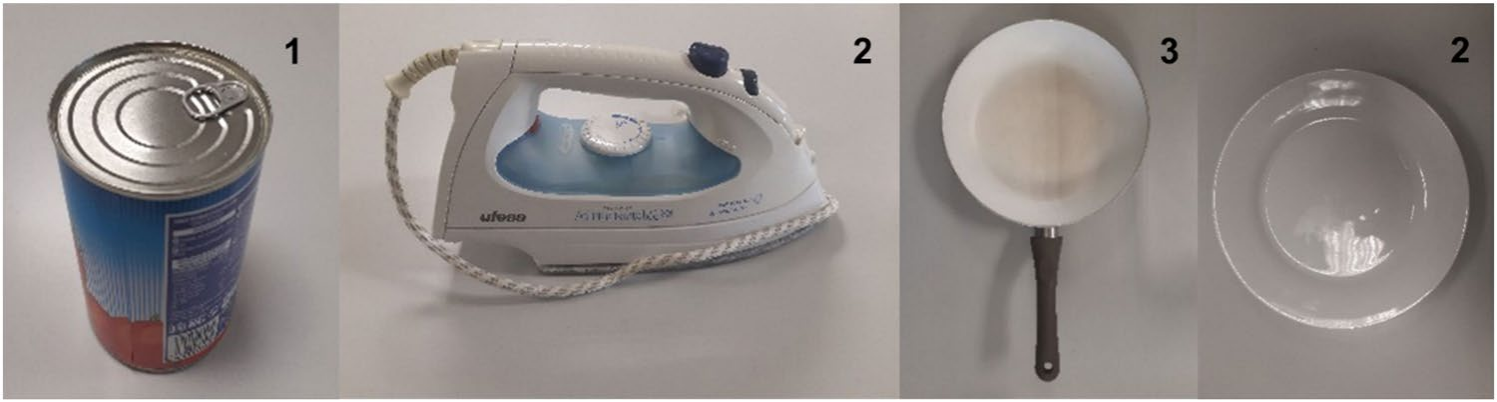
Product orientations considered. 1 = Vertical, 2 = Horizontal transverse, 3 = Horizontal longitudinal. In the special case of dishes and bowls, without a clear grasping axis, “horizontal transversal” orientation was considered. When product orientation was not controlled, it was classified as “4 = other”.

#### PRODUCT_DATA

Substructure containing information regarding product characteristics. Table 3 details the variables, their meaning and codification.

**Table 3:** Fields in PRODUCT_DATA substructure.

VARIABLE	VALUE/LABELLING
*PRODUCT*	ID of the product (accordingly to the guide attached to the dataset)
*WEIGHT*	Product weight (in g)
*SPAN_1*	Main span (in mm) (e.g. diameter of the body of a jar)
*SPAN_2*	Secondary span (in mm) (e.g. diameter of the cap of a jar)
*SHAPE_SPAN_1*	Section shape of main span: (see Fig. 9) 1 = Circular 2 = Rectangular 3 = Circular faceted 4 = Elliptical 5 = Elliptical faceted 6 = Plate 7 = Other
*SHAPE_SPAN_2*	Section shape of secondary span Codified as SHAPE_SPAN_1.
*AD*	Product type: 0 = Not assistive device (common product) 1 = Assistive device

**Fig. 9:**
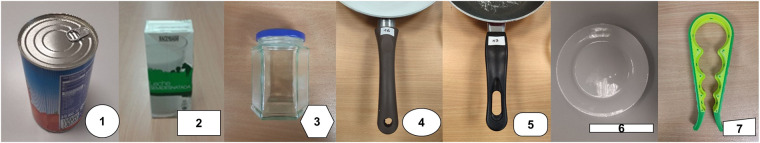
Example of products classified in each section shape group.

### Custom data visualization application (ERGOMOVMUS)

**Fig. 10:**
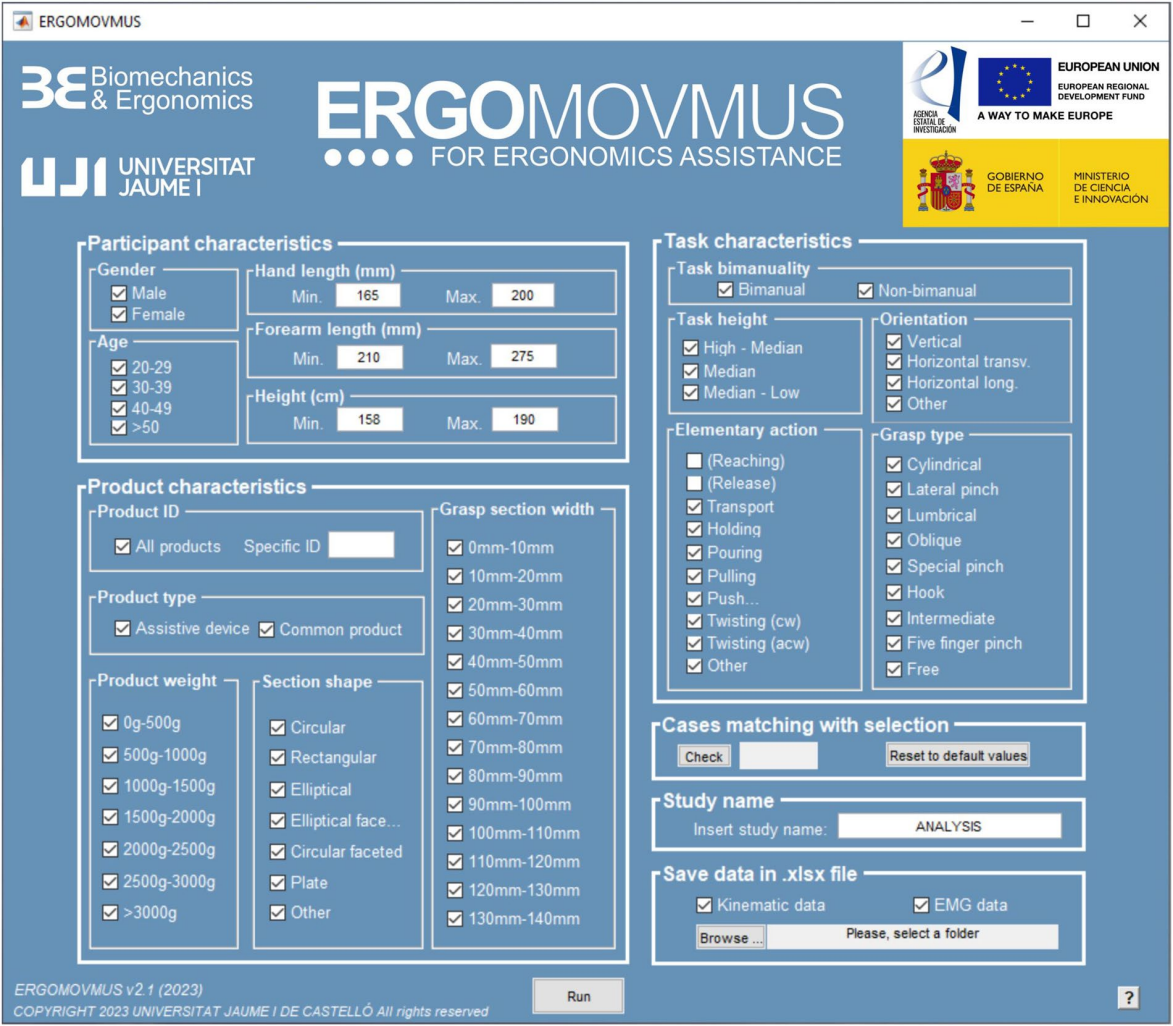
Screenshot of the main menu of ERGOMOVMUS.

A custom data visualization application for ergonomics assistance (ERGOMOVMUS) has been developed using the MATLAB GUIDE environment, in order to easily select different fields regarding participants, task and product characteristics previously detailed. The application is provided with the dataset available at Zenodo^31^, and has several spreadsheets in OOXML Transitional and OpenDocument formats (in.xlsx/.ods) attached: KINEMATIC_DATA.xlsx and sEMG_DATA.xlsx contain a statistical summary of the full recordings of the dataset consisting of 5^th^, 50^th^ and 95^th^ percentiles of each joint kinematics and each spot muscular activation for each subject and elementary task; PARTICIPANTS_DATA.xlsx, TASK_DATA.xlsx and PRODUCT_DATA.xlsx contain the same information as the homonym .mat files of the dataset. The application allows visualizing and saving in an OOXML Transitional format (.xlsx) spreadsheet both kinematic and forearm muscular activation data corresponding to selectable specific characteristics of participants, tasks and products. A screenshot of the main window of the application can be seen in Fig. 10.

## Technical Validation

### Data acquisition

Before and after each experiment phase, participants were asked to randomly move their hands, in order to check that all the gauges were shown active on the virtual model of the CyberGlove software.

After recordings, the number of labels used to divide each task into elementary tasks was checked to be correct, ensuring that no labels were missing. Furthermore, as detailed in previous sections, in order to avoid possible unexpected signal values all kinematic and sEMG data collected were filtered.

### Statistical descriptive analysis of data collected

**Fig. 11:**
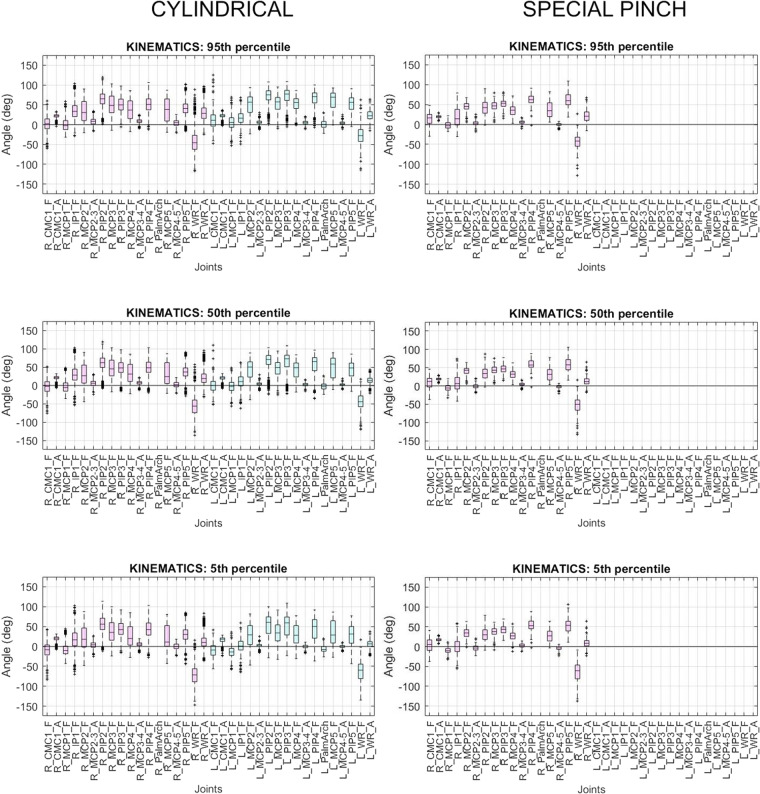
Box and whisker plots of 95^th^, 50^th^ and 5^th^ percentiles of kinematic data collected in tasks requiring cylindrical and special pinch grasp types. Joints and movements labelled as explained in Fig. 6. Forearm spots labelled as explained in the main text. Note that right palmar arch data is not available and does not appear in the plots.

**Fig. 12:**
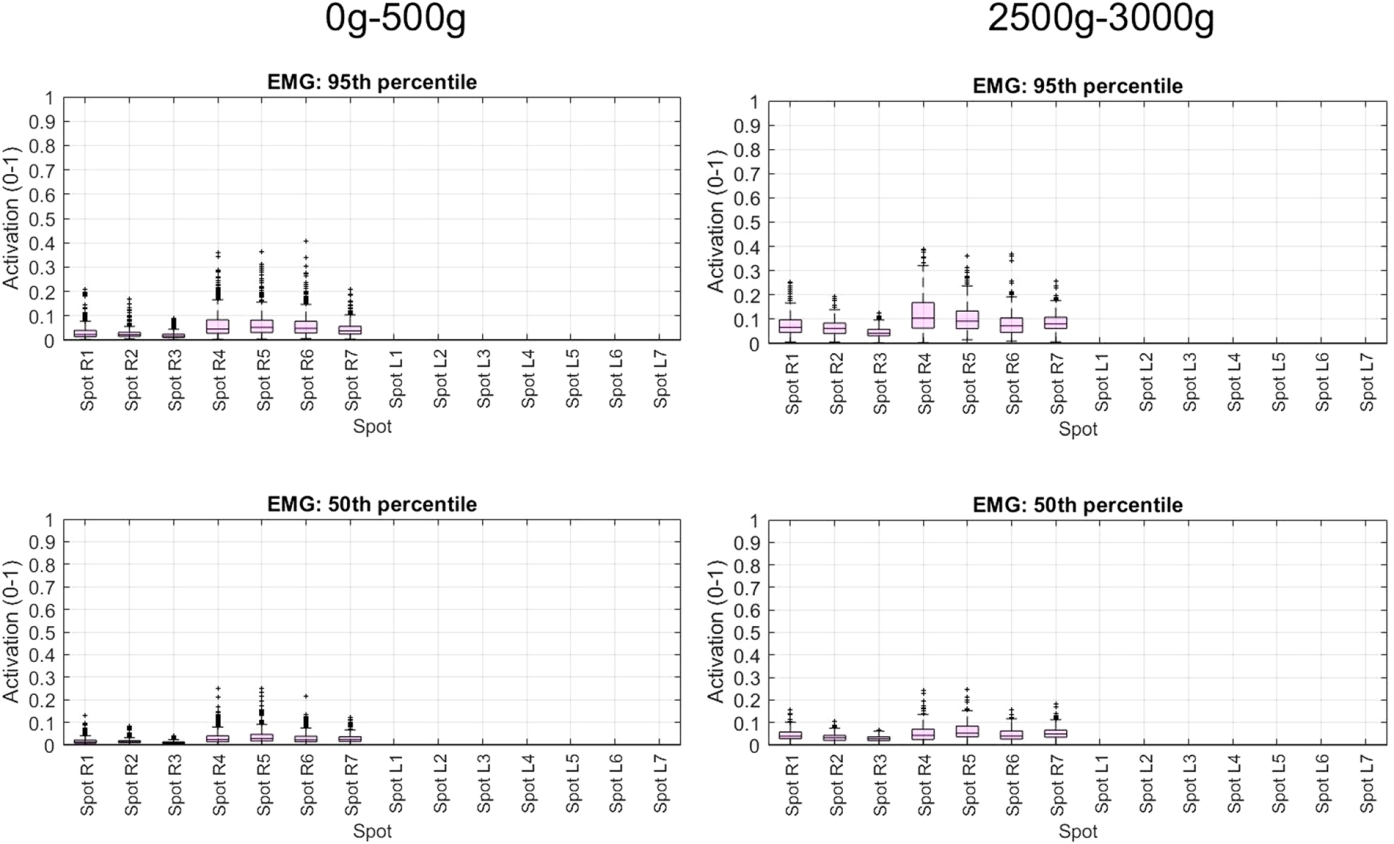
Box and whisker plots of 95^th^ and 50^th^ percentiles of sEMG data collected in transportation tasks using products with weight ≤500 g and 2500g-300g. Forearm spots labelled as explained in the main text.

In order to validate data, 95^th^, 50^th^ and 5^th^ percentiles of joint angles and muscular activation for each participant and elementary task were plotted using box-and-whisker graphs. Kinematic data from two grasp types requiring markedly different postures (cylindrical and special pinch) were plotted for comparison (Fig. 11). Analogously, sEMG data during transportation tasks of products belonging to weight groups 0g–500g and 2500g–3000g were plotted (Fig. 12). It can be observed that the hand posture obtained for each grasp type is different, and values of joint angles are within the ranges obtained in previous studies measuring hand functional range of motion^30,34,35^ or datasets providing hand kinematic data during the performance of different ADLs^6,7,13^. Furthermore, the muscular activation obtained was higher during transportation tasks of products belonging to weight group 2500g–3000g, and the obtained values are aligned with those reported in previous studies where surface electromyography (sEMG) was collected during the performance of a diverse set of ADLs using products with different weights^7,13^.

### Limitations

The use of instrumented gloves may imply some loss of dexterity during the performance of tasks requiring fine manipulation. Nonetheless, this loss of dexterity may not produce a significant effect on mean postures, ranges of motion or motion synergies. Muscle activation has been obtained using surface EMG, which does not allow focusing on a specific muscle, and only from seven specific areas of the forearm where only extrinsic hand muscles are present.

## Usage Notes

These data can be used for several applications, such as machine learning, healthy hand characterization or product design ergonomics. The main strengths of this dataset for these potential uses are its provision of synchronized data of hand kinematics and forearm muscle activation with tagged information regarding participants and task characteristics, and product design features, allowing more specific analyses. Another strength is the variety of products used (105 products) and tasks performed, requiring different elementary actions, grasp types, performance heights and product orientations (161 different tasks, divided into 614 elementary tasks). Finally, the presentation of these data, both as a MATLAB/GNU Octave data structure (*.mat)* and through a custom data visualization application, allows its usability in several applications.

It has to be considered that real food was not used to perform the tasks in order to prevent the gloves from getting stained or wet (all products are appropriately tagged with the corresponding substitute material in the dataset guide file). Therefore, tasks involving food were simulated and might be performed in a slightly different way than when performed with real food.

Finally, it has to be mentioned that velocity of performance of the tasks might be slightly affected by the loss of dexterity and touch sensitivity resulting from the use of the instrumented gloves^36^.

## Data Availability

The custom MATLAB code used to calculate hand anatomical angles is freely available on Zenodo^37^. Its use requires a prior calibration procedure of the data glove, which was developed in previous work^26^.

## References

[1] Sburlea AI, Müller-Putz GR (2018). Exploring representations of human grasping in neural, muscle and kinematic signals. Sci. Rep..

[2] Gustus A, Stillfried G, Visser J, Jörntell H, van der Smagt P (2012). Human hand modelling: kinematics, dynamics, applications. Biol. Cybern..

[3] Nathan, D. E., Johnson, M. J. & Mcguire, J. R. Design and validation of low-cost assistive glove for hand assessment and therapy during activity of daily living-focused robotic stroke therapy. **46**, 587–602 (2021). 10.1682/jrrd.2008.04.0052 PMC8010363 19882493

[4] Bianchi, M., Bohg, J. & Sun, Y. Latest Datasets and Technologies Presented in the Workshop on Grasping and Manipulation Datasets. *arXiv* 1609.02531 10.48550/arXiv.1609.02531 (2016).

[5] Amis AA (1987). Variation of finger forces in maximal isometric grasp tests on a range of cylinder diameters. J. Biomed. Eng..

[6] Roda-Sales, A., Vergara, M., Sancho-Bru, J. L., Gracia-Ibáñez, V. & Jarque-Bou, N. J. Human hand kinematic data during feeding and cooking tasks. *Sci. Data* **6**, (2019). 10.1038/s41597-019-0175-6 PMC6754415 31488844

[7] Jarque-Bou NJ, Atzori M, Müller H (2020). A large calibrated database of hand movements and grasps kinematics. Sci. Data.

[8] Atzori, M. *et al*. *Ninaweb.* http://ninapro.hevs.ch/ (2014).

[9] Gabiccini, M., Stillfried, G., Marino, H. & Bianchi, M. *Hand Corpus.* http://www.handcorpus.org/?p=1578 (2015).

[10] Puhlmann, S., Heinemann, F., Brock, O. & Maertens, M. *Hand Corpus.* http://www.handcorpus.org/?p=1830 (2016).

[11] Atzori M (2014). Electromyography data for non-invasive naturally-controlled robotic hand prostheses. Sci. Data.

[12] Ngeo JG, Tamei T, Shibata T (2014). Continuous and simultaneous estimation of finger kinematics using inputs from an EMG-to-muscle activation model. J. Neuroeng. Rehabil.

[13] Jarque-Bou NJ, Vergara M, Sancho-Bru JL, Gracia-Ibáñez V, Roda-Sales A (2019). A calibrated database of kinematics and EMG of the forearm and hand during activities of daily living. Sci. data.

[14] Furmanek MP, Mangalam M, Yarossi M, Lockwood K, Tunik E (2022). A kinematic and EMG dataset of online adjustment of reach-to-grasp movements to visual perturbations. Sci. Data.

[15] Dwivedi, S. K., Ngeo, J. & Shibata, T. Dataset of Surface Electromyographic (sEMG) Signals and Finger Kinematics. *IEEE Dataport* https://ieee-dataport.org/open-access/dataset-surface-electromyographic-semg-signals-and-finger-kinematics, 10.21227/zbkg-gy95 (2020).

[16] Ansuini C, Giosa L, Turella L, Altoè G, Castiello U (2008). An object for an action, the same object for other actions: Effects on hand shaping. Exp. Brain Res..

[17] McDonald SS, Levine D, Richards J, Aguilar L (2016). Effectiveness of adaptive silverware on range of motion of the hand. PeerJ.

[18] Roda-Sales A, Vergara M, Sancho-Bru JL, Gracia-Ibáñez V, Jarque-Bou NJ (2019). Effect on hand kinematics when using assistive devices during activities of daily living. PeerJ.

[19] Oh SA, Radwin RG (1997). The effects of power hand tool dynamics and workstation design on handle kinematics and muscle activity. Int. J. Ind. Ergon..

[20] Suedbeck JR, Tolle SL, McCombs G, Walker ML, Russell DM (2017). Effects of Instrument Handle Design on Dental Hygienists’ Forearm Muscle Activity During Scaling. J. Dent. Hyg. JDH.

[21] Dong H (2006). The effects of periodontal instrument handle design on hand muscle load and pinch force. J. Am. Dent. Assoc..

[22] Feix T, Bullock IM, Dollar AM (2014). Analysis of human grasping behavior: Object characteristics and grasp type. IEEE Trans. Haptics.

[23] Lee KS, Jung MC (2015). Investigation of hand postures in manufacturing industries according to hand and object properties. Int. J. Ind. Ergon..

[24] Chen X, Li Z, Wang Y (2020). Effect of object and human-factor characteristics on the preference of thumb-index finger grasp type. Ergonomics.

[25] Wu G (2005). ISB recommendation on definitions of joint coordinate systems of various joints for the reporting of human joint motion - Part II: Shoulder, elbow, wrist and hand. J. Biomech..

[26] Gracia-Ibáñez V, Vergara M, Buffi JH, Murray WM, Sancho-Bru JL (2017). Across-subject calibration of an instrumented glove to measure hand movement for clinical purposes. C. Comput. Methods Biomech. Biomed. Eng..

[27] Jarque-Bou NJ, Vergara M, Sancho-Bru JL, Roda-Sales A, Gracia-Ibáñez V (2018). Identification of forearm skin zones with similar muscle activation patterns during activities of daily living. J. Neuroeng. Rehabil..

[28] Hermens HJ, Freriks B, Disselhorst-Klug C, Rau G (2000). Development of recommendations for SEMG sensors and sensor placement procedures. J. Electromyogr. Kinesiol..

[29] Konrad, P. *The ABC of EMG: A Practical Introduction to Kinesiological Electromyography*. *Version 1.0* (Noraxon Inc. USA, 2005).

[30] Gracia-Ibáñez V, Vergara M, Sancho-Bru JL, Mora MC, Piqueras C (2017). Functional range of motion of the hand joints in activities of the International Classification of Functioning, Disability and Health. J. Hand Ther..

[31] Roda-Sales A (2023). Zenodo.

[32] Kapandji, A. I. *The Physiology of the Joints. Volume I: Upper Limb*. (Editorial Médica Panamericana, 1998).

[33] Brand, P. W. & Hollister, A. M. *Clinical Mechanics of the Hand*. (Mosby Publishing, 1999).

[34] Hume MC, Gellman H, McKellop H, Brumfield R (1999). Functional Motion of the joints of the hand. Am. Acad. Orthop. Surg..

[35] Bain GI, Polites N, Higgs BG, Heptinstall RJ, McGrath AM (2015). The functional range of motion of the finger joints. J. Hand Surg. Eur. Vol..

[36] Roda-Sales, A., Sancho-Bru, J. L., Vergara, M., Gracia-Ibáñez, V. & Jarque-Bou, N. J. Effect on manual skills of wearing instrumented gloves during manipulation. *J. Biomech*. **98**, (2020). 10.1016/j.jbiomech.2019.109512 31767287

[37] Gracia-Ibáñez V, Jarque-Bou NJ, Roda-Sales A, Vergara M, Sancho-Bru JL (2019). Zenodo.

